# Plant Endophytic Fungus Extract ZNC Improved Potato Immunity, Yield, and Quality

**DOI:** 10.3389/fpls.2021.707256

**Published:** 2021-09-16

**Authors:** Juan Cao, Baoyou Liu, Xinning Xu, Xiaoying Zhang, Changxiang Zhu, Yang Li, Xinhua Ding

**Affiliations:** ^1^State Key Laboratory of Crop Biology, Shandong Provincial Key Laboratory for Biology of Vegetable Diseases and Insect Pests, College of Plant Protection, Shandong Agricultural University, Tai’an, China; ^2^Shandong Pengbo Biotechnology Co., Ltd., Tai’an, China; ^3^Yanzhou Agricultural Technology Extension Center, Yanzhou, China; ^4^Yantai Academy of Agricultural Sciences, Yantai, China; ^5^College of Life Sciences, Yantai University, Yantai, China

**Keywords:** plant immune inducer, ZNC, plant growth, plant immunity, *Phytophthora infestans*

## Abstract

Endophytic fungi play an important role in plant survival and reproduction, but the role of their metabolites in plant growth and immunity, as well as in crop quality formation, is poorly understood. Zhinengcong (ZNC) is a crude ethanol extract from the endophytic fungus *Paecilomyces variotii*, and previous studies have shown that it can improve the growth and immunity in *Arabidopsis thaliana*. The aim of the study was to reveal the trade-off balance between plant growth and immunity by evaluating the mechanisms of ZNC on potato growth, yield, and priming immunity against the oomycete *Phytophthora infestans* indoors and in the field. ZNC maintained a good balance between plant growth and resistance against *P. infestans* with high activity. It induced the reactive oxygen species (ROS) production, promoted plant growth, yield and quality parameters, enhanced the expression of indoleacetic acid (IAA) related genes, and increased the absorption of nitrogen from the soil. Moreover, the plant endophytic fungus extract ZNC stimulated the pathogen-associated molecular pattern (PAMP) triggered immunity (PTI) pathway and contributed to the ZNC-mediated defense response. Two years of field trials have shown that irrigation with ZNC at one of two optimal concentrations of 1 or 10ng/ml could significantly increase the output by 18.83% or more. The quality of potato tubers was also greatly improved, in which the contents of vitamin C, protein, and starch were significantly increased, especially the sugar content was increased by 125%. Spray application of ZNC onto potato plants significantly reduced the occurrence of potato blight disease with 66.49% of control efficacy at 200ng/ml and increased the potato yield by 66.68% or more in the field. In summary, plant endophytic fungus extract ZNC promoted potato immunity, yield, and quality and presented excellent potential in agricultural applications.

## Introduction

Potato (*Solanum tuberosum* L.) is one of the most important staple crops worldwide, preceded only by rice and wheat, playing an essential role in the sustainable food supply and decreasing poverty and malnutrition (Robertson et al., 2018). Potato late blight, caused by the oomycete *Phytophthora infestans*, is a devastating disease (Jiang et al., 2018). Despite the large amounts of chemical pesticides used by growers, this disease is still a major cause of potato yield loss worldwide (Arora et al., 2014). In addition, the long-term and intensive use of chemical fertilizers is damaging to soil quality and the natural environment, which further threatens human health through the food chain (Sun et al., 2018). Recently, an increasing number of growers and consumers around the world have raised awareness on the importance of effective and sustainable food production (Guyer et al., 2015). Therefore, it is essential to find environment-friendly agro-chemicals with dual functions in crop nutrient uptake and disease resistance management, which ensures the sustainable development of agricultural products.

The challenges of an increasing population, malnutrition, development of resistant pathogens, and ecological deterioration have put forth an ever-increasing demand for new, durable, effective, and safe agro-chemicals to combat plant diseases, promote food quality, enhance crop yield, and improve living standards. More than 49% of the new medicines approved by the United States Food and Drug Administration (FDA) are derived from natural products or their derivatives (Patil et al., 2016). Likewise, there is renewed interest in organism-derived agro-chemicals with minimal or no side effects, one of the potential alternatives to synthetic chemical pesticides and fertilizers. Approximately 75% of the existing antimicrobials are produced from living organisms (Patil et al., 2016). Among them, microorganisms are one of the most important antimicrobial sources and can be isolated from natural habitats, including the soil. By one or more direct or indirect patterns, plant growth-promoting rhizobacteria (PGPR) activate plant growth (Kalam et al., 2017). Endophytic strains live inside plant tissues and maintain mutualistic and close relationships with their hosts without inducing any apparent infection or disease symptoms (Li et al., 2018). Endophytic fungi could assist plants by synthesizing plant growth regulators and supplementing plant nutrient acquisition. Twelve endophytic fungi isolated from *Vanda critata* showed growth-promoting activity in *Cymbidium aloifolium* protocorms, while the endophytic fungus *Fusarium oxysporium* CVS4 was found to have high plant growth-promoting activity (Chand et al., 2020).

Moreover, these plant growth-promoting-like effects appear to involve indirect stimulation, such as competition for space and nutrients in the rhizosphere, production of antagonistic substances to suppress deleterious microorganisms or root phytopathogens, and/or induction of plant systemic resistance against many plant pathogens, commonly referred to as plant immune inducers (Zhou et al., 2016). Three plant growth-promoting fungi, *Trichoderma asperellum* SKT-1, *Fusarium equiseti* GF18-3, and *Penicillium simplicissmum* GP17-2, were found to show strong effects both in enhancing onion growth and reducing the disease severity of onion white rot disease (Elsharkawy and El-Khateeb, 2019). Endophytic fungal strains can also prevent plants from attack by various insect pests through excreting bioactive metabolites to inhibit pathogen growth directly or promote plant resistance (Reinhold-Hurek and Hurek, 2011). Four endophytic strains of *Bacillus subtilis* var. *amyloliquefaciens* presented significantly high inhibition of rice bacterial leaf blight (Nagendran et al., 2014). Endophytic *B. subtilis* Lu144 strongly decreased the disease incidence of bacterial wilt of mulberry (Ji et al., 2010). In addition, endophytic *B. subtilis* E1R-j demonstrated a strong antifungal activity against *Gaeumannomyces graminis* var. *tritici* (Liu et al., 2009).

However, the application of endophytes is still limited for several reasons. On the one hand, the specific mechanisms of these endophytes remain unknown as well as details like the suitable growth conditions of many endophytes (Ownley et al., 2010). On the other hand, the practical application of living endophytes requires specific application techniques to acquire the best results under field conditions. An alternative method is to apply the extracts of these endophytic organisms to achieve high activity without limitations from the environmental conditions and hosts, similar to the methods used for conventional agricultural chemicals (Monjil et al., 2015). The biological control agent zhinengcong (ZNC) is the extract of endophytic *Paecilomyces variotii* isolated from *Hippophae rhamnoides*, which is an epiphytic orchid that possesses a great diversity of endophytic fungi (Lu et al., 2019). ZNC promotes plant growth and enhances plant resistance against bacteria and viruses (Lu et al., 2019; Peng et al., 2020). However, it has not yet been tested against economically important oomycetes in potato. In this paper, we used potato and *P. infestans* as materials to study the ZNC role in plant growth, yield, and immune activities indoors and in the field. ZNC triggered the activity for a series of plant growth promoting and defense responses, such as indoleacetic acid (IAA) accumulation, reactive oxygen species (ROS) burst, and the activation of PTI pathway. It was surprising that ZNC could significantly increase potato yield as well as disease resistance against potato late blight in field experiments. This study first demonstrates that ZNC can maintain the balance between plant growth and resistance in potato and provides a new bioreagent to develop sustainable and cost-effective strategies for potato production and biological prevention of potato late blight.

## Materials and Methods

### Experiment Materials and Design Indoors

Zhinengcong, the crude ethanol extract of *P. variotii* SJ1, was provided by Shandong Pengbo Biotechnology Co., Ltd. (Tai’an, Shandong, China; Lu et al., 2019) and could be diluted in water. The SJ1 strain was deposited in China General Microbiological Culture Collection Center^1^ with the number CGMCCNO.10114. The SJ1 (SJ1) hyphae obtained by three-stage liquid fermentation was filtered with a 300-mesh filter cloth under pressures of 0.2 and 0.4MPa respectively, and mixed with the same volume of 95% alcohol (Wang et al., 2021). The mixture was sealed for 2weeks to extract ZNC. The mixture was injected into a 500-L tank and the experiment was performed at 25°C with a stirring speed of 1,000g/min and a frequency of 20.0kHz. Then, the mycelium residue was abolished with a filter press. After drying under vacuum at 40°C, ZNC was obtained (Wang et al., 2021). As for quality control, enzyme-linked immunosorbent assay and chromatographic fingerprinting were used to evaluate the similarity and specificity of different batches (Wang et al., 2021). The dried ZNC pellet could be dissolved completely in 20% alcohol, and the concentration of stock solution was 5mg/ml. When used, the ZNC stock solution is diluted with water.

Favorita potato tubers were sown in pots after being applied to break dormancy of tuber sprouting by using 10mg/kg gibberellin. Each pot contained 75g sterilized Pindstrup substrate peat (pH 5.6–6.4, size 0–10mm; Pindstrup Mosebrug A/S, Ryomgaard, Denmark). Potato sprouts were grown in the incubation chamber under 70% relative humidity and 12h/12h for the day and night, respectively, at 23°C. Potato sprouts were root irrigated with 0, 1, 10, or 100ng/ml ZNC twice at day 0 and 25, and 10ml of the ZNC solution was applied to each pot. To compare the growth-promoting effects of ZNC on potato at 1, 10, and 100ng/ml, the growth parameters of plant height, leaf area of parietal lobules of the third and fourth leaves, stem diameter, root length, and root weight were recorded at day 20, 30, 40 and 60, respectively.

### Culture Preparation and Spore Suspension of *Phytophthora infestans*

The plant pathogenic oomycete *P. infestans* HLJ was cultured on sterile rye medium and kept in an incubator at 18°C in the dark for 14days (Wang et al., 2019). The sporangia of *P. infestans* were scraped from the mycelial surface using 5ml ddH_2_O, filtered using three layers of gauze, collected into 10ml centrifuge tubes, and placed on ice for 2.5h to induce zoospore release from the sporangia. The spore density was counted by a hemocytometer under an optical microscope, and the concentration was adjusted to 3.4–6.8×10^5^ zoospores/ml with ddH_2_O.

### Virulence Assays

Detached leaves taken from 40-day-old Favorita potatoes were placed upside down on moistened filter paper in a 25-by-25-cm plate and inoculated with 20μl of sporangia and zoospore suspension using a pipette. The above treated samples were then cultured in a constant temperature incubator at 18°C and 95% RH with 16h of weak light. The whole potato plants were evenly sprayed with the spore suspension and then placed in a greenhouse at a relative humidity of 95% or more at 18–22°C. The disease index was counted at day 5 or 7 using the following standards: 0=no disease, 1=1–25%, 2=25–50%, 3=50–75%, and 4 ≥75%, where the percentages were the proportion of diseased area to total area. The experiment was repeated four times independently with three replicates each.

### Histochemical Staining of ROS

The ZNC solution (0, 1, 10, and 100ng/ml) was sprayed on 40-day-old Favorita potato leaves to runoff. Every treatment contained six potato plants. After 2h, three leaves were subjected to nitroblue tetrazolium (NBT) and 3,3-diaminobenzidine (DAB) staining after 2h to evaluate ROS production (Lu et al., 2019).

Detached leaves were immersed in sodium azide solution (1% M/V), vacuum-infiltrated for 30min and shifted into 0.5mg/ml NBT solution followed by vacuum infiltration for 30min. NBT reacted with O^2−^ forming a dark blue water-insoluble complex substance. The excess dye solution was washed away by boiling in ethanol before imaging.

The *in situ* detection of hydrogen peroxide (H_2_O_2_) in detached leaves was determined by DAB staining. The leaves were immersed in 1mg/ml DAB solution by gentle vacuum infiltration for 30min while the container was covered with aluminum foil. The stained leaves were washed three times with ddH_2_O and kept under light for 8h at 28°C. Following incubation, the DAB staining solution was replaced with boiling ethanol. DAB was oxidized by H_2_O_2_ in the presence of some haem-containing proteins to generate a dark brown precipitate in leaves. Quantitative analysis of the histological staining and fluorescence intensity of ROS was carried out using ImageJ under the same program.

### RNA Extraction and Quantitative Real-Time PCR

Plant samples were taken from 40-day-old leaves from Favorita potato plants irrigated with 0, 1, 10, and 100ng/ml ZNC to analyze gene transcriptional level, respectively. The third and fourth leaves were collected with three biological replicates. All tubes and solutions are RNase-free, and total RNA was extracted from treated leaves with TRIzol reagent (Invitrogen, Carlsbad, CA, United States) according to the manufacturer’s instruction. A total amount of 2μg of extracted RNA with a ratio of OD260/OD280 of 1.95–2.05 was reversely transcribed into cDNA using the iScript cDNA Synthesis Kit (Bio-Rad). Real-time analysis for genes of interest was performed with SYBR Green Master Mix (Thermo Fisher Scientific, MA, United States) following the instructions. qPCRs with three technical repeats for every sample were carried out using a QuantStudio™ Flex Real-Time PCR System. The relative expression values of mRNA were normalized to the housekeeping gene *StEF1a* and *RPN7*. The fold change values of quantitative were defined by the equation 2^−ΔΔ*C*t^. The melting curve for each gene of interest was analyzed to validate the specificity of every amplification. The sequences of the primers used in this study are designed by the Primer Premier 5 software and listed in Supplementary Table 1.

### RNA-Sequencing Data Analysis

Plant samples were the third and fourth leaves of 40-day-old Favorita potato plants irrigated with 0, 1, 10, and 100ng/ml ZNC, respectively, and the experiment was performed independently three times. The total RNA was extracted from treated leaves with Plant RNA Kit (Omega, GA, United States) and used for RNA-sequencing by Beijing Genomics Institute. If the RIN score of an RNA sample was more than 7, it was tested by the Wuhan Genomic Institution to yield valuable data. Readings with low quality, contaminated linker and high nitrogen content of unknown bases were filtered out. The filtered data were called clean reads and compared to the reference genome of *Solanum tuberosum* in GCF_000226075.1_SolTub_3 by Hierarchical Indexing for Spliced Alignment of Transcripts (HISAT) software. First, HISAT used the global Ferragina-Manzini (FM) index to anchor the position of the partial sequence of each read on the genome. Then, the local index of these alignment positions were used to align the remaining sequences of each read to extend the alignment area. StringTie was used to reconstruct the transcript of each sample. Cuffmerge was used to integrate the reconstruction information of all samples, and then Cuffcompare was used to compare the integrated transcript with the reference annotation information. Thus, new transcripts were predicted, and SNP & InDel and differentially spliced gene were detected. After obtaining the new transcript, the new transcripts with protein coding potential were added to the reference gene sequence to form a complete reference sequence, and then the gene expression level was calculated by RNA-Seq by Expectation-Maximization (RSEM) software (version 1.2.31). Finally, the differentially expressed genes between different samples for multiple samples were detected and based on fragments per kilobase of transcript per million fragments mapped (FPKM; Zhao et al., 2021). The values of relative gene expression were represented by log10(FPKM+1), and differential expression analysis adopted log2FoldChange. In-depth cluster analysis and functional enrichment analysis on the differentially expressed genes were performed. The raw RNA sequence data have been registered at the NCBI Sequence Read Archive with the accession number PRJNA681705.

### Gene Ontology Enrichment Analysis

Gene Ontology (GO) enrichment analysis is that the GO function items in candidate genes are significantly enriched compared with the entire genetic background of the species. The analysis first maps all candidate genes to each item in the GO database.^2^ Then, the “GO::TermFinder” software^3^ was used to calculate the number of genes in each term, and the phyper function in the R software was used to perform an enrichment analysis test to find GO term compared with the background of all genes in this species. Thus, the candidate genes were significantly enriched in term. Moreover, FDR correction was performed on the *p* value. Usually, the function with *Q* value ≤0.05 is regarded as significant enrichment.

### Measurement of Total Nitrogen

For total nitrogen detection, ZNC-treated potato leaves were collected at day 60, oven-dried at 70°C to constant weight and then ground into homogenized powder. Approximately 150mg of each sample was digested with H_2_SO_4_–H_2_O_2_ at 350°C and 280°C for nitrogen quantification according to the previous method (Sun et al., 2018).

### Experiments With Root Irrigation and Foliar Spray Application in the Field

Field experiments using Favorita potato cultivar were conducted in 2019 and 2020 at two locations in Shandong Province, China. The experimental sites had a long history of potato cultivation. (1) The root irrigation experiment was conducted in Yanzhou County (35°53'N, 116°77'E), Jining City, where plants were subjected to common agricultural crop protection schemes to prevent natural late blight infection. The potatoes under greenhouse conditions were sown in January and harvested in May, 2019 and 2020. To identify how ZNC influences potato growth, four treatments of ZNC at 0, 1, 10, and 100ng/ml were applied to potato plants with a planting density of 57,000 plants per hm^2^. Twelve plots were designed according to a random complete block design (RCBD) arrangement with three replicate plots per treatment. ZNC was applied twice, once at day 45 after being planted and once at day 70 of the flowering stage by 200ml per plant. To avoid cross contamination, 1m intervals were left between blocks. In each plot, 10 plants were randomly selected before harvest for measurement of potato growth indices with a straight ruler and Vernier calipers and for evaluation of potato yield by using the following grading standards: large potatoes were more than 250g, medium potatoes were 50–250g, and small potatoes were less than 50g. The quality of potatoes was tested by Shandong Yihui Testing Technology Co., Ltd. according to standard methods (Wang et al., 2017). (2) The foliar spray experiment was conducted at the Science and Technology Service Industry Pilot Zone (35°95'N, 117°38'E), Tai’an City. In this experimental site, natural late blight disease was endemic. The potatoes under field conditions were sown in March and harvested in July, 2020. Six treatments of ZNC at 0, 10, 25, 50, 100, and 200ng/ml were sprayed on potato leaves at a planting density of 60,000 plants per hm^2^. Eighteen plots were designed according to random block arrangements with three replicate plots per treatment. One plot of 60m^2^ was spayed by 4kg of ZNC solution. The assays were conducted following the methods mentioned above.

### Statistical Analysis

All data were processed using GraphPad Prism 5.0. One-way ANOVA analysis and DESeq2 method were used to analyze the obtained data from indoor and field experiments and to determine significant differences between treatments.

## Results

### Trace Amounts of ZNC Promote the Growth of Potato Roots and Plants in Growth Chamber

**Figure 1:**
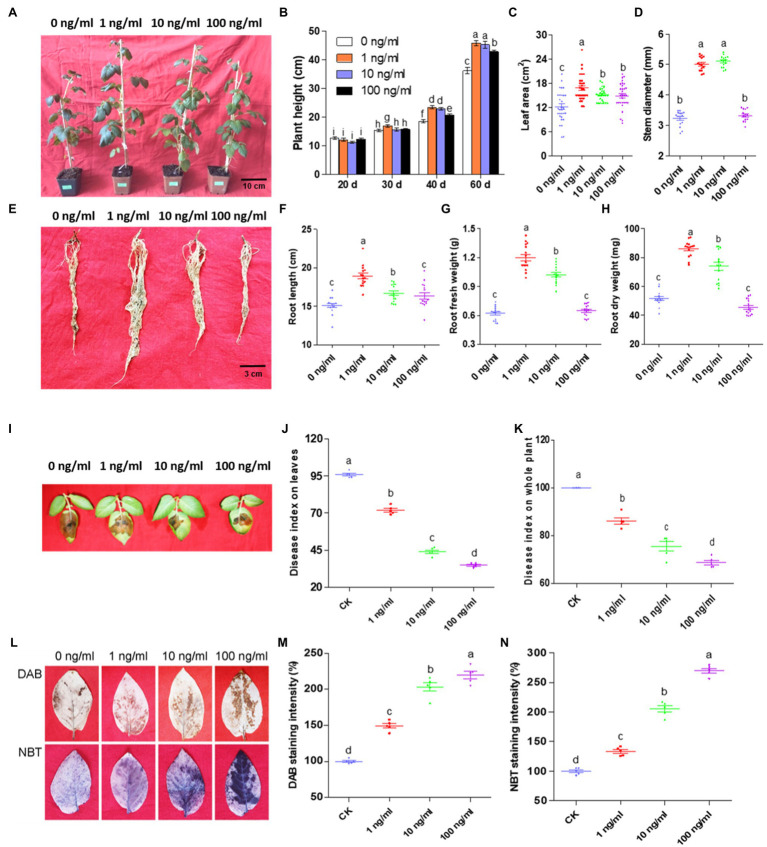
Zhinengcong (ZNC) promotes potato growth and late blight resistance corresponds with ROS accumulation. **(A)** Potatoes at day 60 after cultivation. Scale bars=10cm. **(B)** Statistical analysis of the aerial part at day 20, 30, 40, and 60. Scale bars=3cm. Data are shown as the mean (15)±SD. **(C)** Potato leaf area at day 60. Data are shown as the mean (30)±SD. **(D)** Stem diameter at day 60. Data are shown as the mean (15)±SD. **(E)** Roots at day 80. Scale bars=3cm. **(F–H)** Root length, fresh root weight, and dry root weight at day 80. Data are shown as the mean (15)±SD. **(I)** Potato leaves inoculated with *Phytophthora infestans* and treated with ZNC at day 40. **(J)** The disease index of detached leaves analyzed on the 5th day after inoculation. Data are shown as the mean (5)±SD. **(K)** The disease index of whole plants analyzed on the 7th day after inoculation. Data are shown as the mean (5)±SD. **(L)** Hydrogen peroxide (upper panel) and superoxide accumulation (lower panel) in potato leaves treated with 0, 1, 10, and 100ng/ml ZNC at 2h post treatment (hpt). **(M,N)** Quantification of ROS staining intensity by DAB **(M)** and NBT **(N)** in potato leaves treated with 0, 1, 10, and 100ng/ml ZNC at 2h post treatment (hpt). Data are shown as the mean (5)±SD. In the ZNC evaluation experiments of plant growth **(A–H)** and plant resistance **(I–K)**, potato sprouts were root-irrigated with 0, 1, 10, or 100ng/ml ZNC twice at day 0 and 25. In the ROS accumulation experiments **(L–N)**, Favorita potato leaves were sprayed to runoff with 0, 1, 10, or 100ng/ml ZNC. Different letters represent significant differences compared with the values at 0ng/ml ZNC (*p*<0.05, based on one-way ANOVA analysis).

To evaluate ZNC, the growth indices of potato plants were recorded at day 20, 30, 40, and 60. As shown in Figures 1A,B, 1ng/ml ZNC significantly promoted potato height by 10, 27, and 28% compared with 0ng/ml at day 30, 40, and 60, respectively, while 10ng/ml ZNC promoted potato height at day 40 and 60. However, the plant height-promoting effects of 100ng/ml ZNC were significantly lower than those of 1 and 10ng/ml ZNC. Potato leaf area and stem diameter at day 60 were measured by a leaf area meter and Vernier caliper, respectively (Figures 1C,D). The ZNC treatments (1, 10, and 100ng/ml) significantly increased the leaf area by 36.76, 27.04, and 22.25%, respectively. Both 1 and 10ng/ml ZNC markedly enhanced the stem diameter by 67%, while 100ng/ml ZNC increased the stem diameter slightly without a significant difference compared with 0ng/ml. Additionally, 1 and 10ng/ml ZNC significantly increased the potato root length and root weight, which were measured at day 80, especially for the 1ng/ml ZNC treatment (Figures 1E–H). These results showed that low concentrations (10^−8^–10^−9^) of ZNC could promote potato growth parameters, including height, leaf area, stem diameter, root length, and root weight.

### ZNC Improves Plant Resistance to Potato Late Blight Accompanied by ROS Accumulation

Our previous study demonstrated that ZNC could enhance plant resistance to bacterial and viral diseases. To test whether ZNC improves potato resistance to oomycete diseases, we detected the effect of ZNC on *P. infestans* infection. Compared with 0ng/ml, ZNC significantly inhibited the occurrence of potato late blight on detached leaves (Figures 1I,J) and on whole plants (Figure 1K). The disease index decreased with increasing ZNC concentrations, especially 100ng/ml, suggesting that ZNC improves disease resistance to potato late blight.

Since ZNC prevented potato late blight (Figures 1I–K) and 1,000ng/ml ZNC did not inhibit the mycelial growth, sporangia number, or germination rate of *P. infestans* sporangia on rye medium (Supplementary Figure 1), ZNC may serve as a inducer to upregulate the defense responses of plants. To prove this hypothesis, ROS levels in potato treated with ZNC were analyzed. The production of hydrogen peroxide and superoxide was detected by DAB and NBT staining, and an increase in brown and purple staining, respectively, was observed 2h after treatment (Figure 1L). Quantitative analysis also showed that the staining intensity under the ZNC treatment increased 30–100% compared with that under 0ng/ml (Figures 1M,N). These results verified that ZNC stimulated the accumulation of ROS in potato leaves and disease resistance.

### RNA-Seq Showed Differences in Gene Expression After ZNC Treatment

To further reveal the molecular mechanisms of ZNC in promoting potato growth and disease resistance, RNA-seq was performed in ZNC-treated and non-treated potatoes through the irrigation method. In this RNA-seq project, a total of 12 samples were tested using the DNBSEQ platform (Beijing Genomics Institute), and each sample produced an average of 10.51Gb of data. More than 0.8 billion clean reads were generated, and an average of 70.09 million clean reads were mapped on the potato genome with an average matching rate of 80.49%, representing an average of 27,264 genes expressed in each sample (Supplementary Table 2). We focused more on the difference between ZNC-treated and nontreated plants in the expression patterns of these upregulated and downregulated genes. Compared with 0ng/ml, a total of 530, 1,176, and 1,647 upregulated genes and 397, 413, and 792 downregulated genes were detected after 1, 10, and 100ng/ml ZNC application (*p*<0.05), respectively (Supplementary Figures 2A–E). From the GO function enrichment analysis of the differentially expressed genes (DEGs) between 0ng/ml and three ZNC treatments, it could be seen that ZNC mainly affected genes with GO annotations: plant hormone signal transduction, microbe-associated molecular patterns (MAPK) signaling pathways, plant–pathogen interaction, phenylpropanoid biosynthesis, and starch and sucrose metabolism (Supplementary Table 3).

### ZNC Irrigation Treatment Activates PTI Pathway in Potato

**Figure 2:**
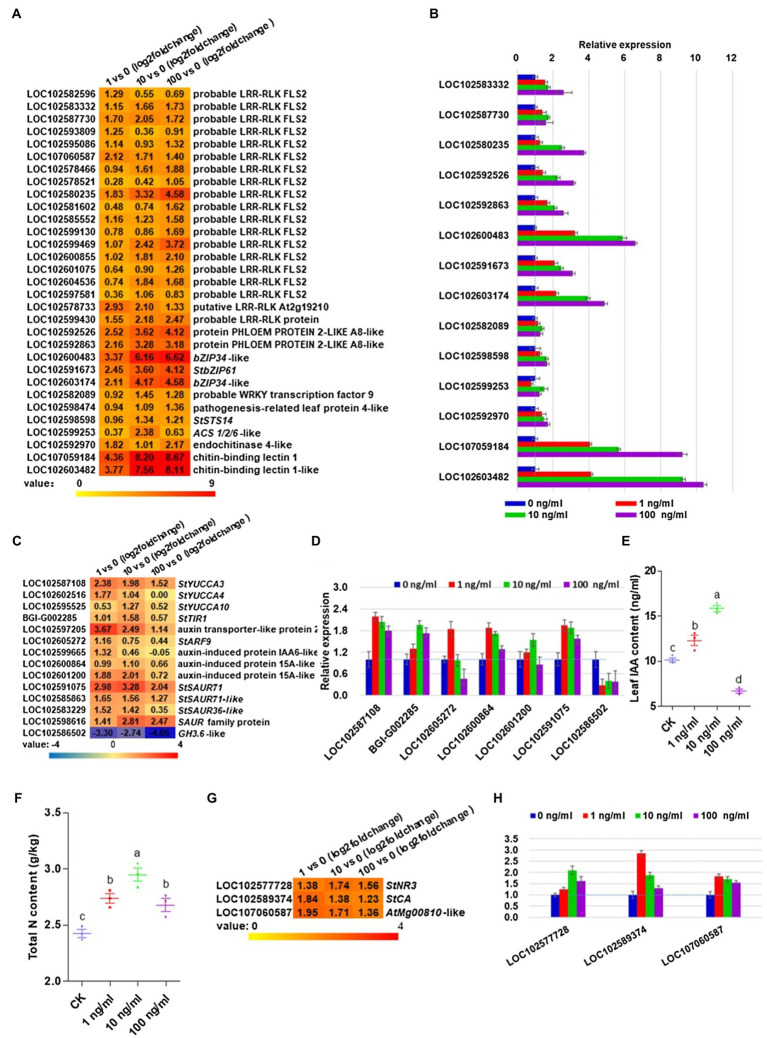
Zhinengcong irrigation activated the PTI pathway and promoted the accumulation of indoleacetic acid (IAA) and nitrogen in potato. **(A)** Genes in PTI pathway induced at the transcriptional level in potato leaves treated with 1, 10, and 100ng/ml ZNC. **(B)** qRT-PCR analysis of genes in PTI pathway subjected to ZNC treatment. **(C)** Upregulated genes involved in auxin in potato leaves treated with ZNC. **(D)** qRT-PCR analysis of IAA-related genes subjected to ZNC treatment. **(E)** Total IAA of potato at day 40 following treatment with ZNC. **(F)** Total nitrogen content of potato treated by ZNC. **(G,H)** Upregulated genes involved in nitrogen metabolism in potato leaves treated with ZNC by RNA-seq. Data are shown as the mean (3)±SD. Different letters represent significant differences compared with the values at 0ng/ml (*p*<0.05 based on one-way ANOVA analysis).

To further investigate which signaling pathway could be induced by ZNC and why ZNC could lead to improved resistance to late blight, we further analyzed RNA-seq data. Plant hormone signal transduction, MAPK signaling pathway, plant–pathogen interaction, phenylpropanoid biosynthesis, and starch and sucrose metabolism were the top five KEGG pathways associated with the highest gene expression changes in all three treatments (Supplementary Table 3). For the MAPK signaling pathway and plant-pathogen interaction, we found that some genes were induced at the transcriptional level with *p*<0.05, including 19 probable leucine-rich repeat receptor like kinase (LRR-RLK) FLAGELLIN-SENSING 2 (FLS2) proteins, two PHLOEW PROTEIN 2-LIKE A8-like proteins belonging to the mitogen-activated protein kinase kinase (MEKK1) protein catalog, four transcription factors, two pathogenesis leaf protein 4-like proteins (LOC102598474 and LOC102598598), one 1-aminocyclopropane−1-carboxylic acid synthase (ACS) 1/2/6-like protein and three substances of chitin signaling (Figure 2A). Interestingly, two MEKK1 proteins and one pathogenesis leaf protein 4-like protein (StSTS14, PR1, and LOC102598598) may be involved in the ROS burst. Two chitin-binding lectin 1 genes showed a 3.77–8.67-fold increase in potato leaves treated with different concentrations of ZNC. To confirm these results, we analyzed the aforementioned genes with qRT-PCR and obtained the same results normalized to the housekeeping gene *StEF1a* (Figure 2B) and *RPN7* (Supplementary Figure 3). Collectively, the above data demonstrate that ZNC could enhance resistance to potato late blight, probably *via* stimulation of the PTI pathway by ZNC.

### ZNC Treatment Promotes Auxin Biosynthesis in Potato

The above results showed that 10^−8^–10^−9^ng/ml of ZNC could promote potato growth parameters, allowing us to explore what gene expression is involved. The indole-3-pyruvic acid (IPA) pathway is the common IAA biosynthesis pathway in plants. From RNA-seq data, three flavin monooxygenase (YUCCA) flavin monooxygenases (StYUCCA3, StYUCCA4, and StYUCCA10), which convert IPA into IAA, were induced by ZNC. The auxin-binding receptor *StTIR1* was upregulated 1.01–1.58-fold following treatment with 1 and 10ng/ml ZNC. ZNC activated auxin transporter-like protein 2 (LOC102597205) and auxin response factor genes (StARF9 and LOC102605272). The ZNC-induced genes in potatoes also include three auxin-induced proteins and four small auxin upregulated (*SAUR*) genes (Figure 2C). Meanwhile, one IAA-amido synthase (*GH3.6*-like, LOC102586502) was dramatically repressed above 2.74-fold (Figure 2C). The quantitative real-time PCR results were consistent with the transcriptomic analysis results normalized to the housekeeping gene *StEF1a* (Figure 2D) and *RPN7* (Supplementary Figure 3). We then tested the IAA content of potato leaves treated with ZNC at day 40, compared with 0ng/ml, 1 and 10ng/ml ZNC treatments increased IAA levels by 18.27 and 45.45%, respectively, while 100ng/ml ZNC reduced IAA levels by 46.16% (Figure 2E). These results indicated that ZNC promoted plant growth probably by promoting the auxin contents in plants at concentrations of 1 and 10ng/ml.

### ZNC Promotes Nitrogen Accumulation and Related Gene Expression in Potato

Nitrogen is not only an essential nutrient for plant growth and development but also provides signals that can trigger downstream pathways, such as gene expression, metabolism, physiology, growth, and developmental processes in plants. Thus, we further measured the total nitrogen content and found that the ZNC treatment significantly increased the total nitrogen content of potato leaves (Figure 2F), which may be related to potato growth traits at 1 and 10ng/ml (Figure 1). To further determine why ZNC promoted potato uptake of nitrogen, the differentially expressed genes were analyzed. Three genes involved in nitrogen metabolism, *StNR3* (LOC102577728), *StCA* (LOC102589374), and *AtMg00810*-like (LOC107060587), were upregulated (Figure 2G). qRT-PCR data were normalized to the housekeeping gene *StEF1a* (Figure 2H) and *RPN7* (Supplementary Figure 3), and showed that the above genes had similar expression patterns to the RNA-sequencing results.

### Irrigation With ZNC Improved Potato Growth and Yield at Ultralow Concentrations

The above indoor experiment results showed that ZNC has significant application potential. To further evaluate the effects of ZNC on potatoes, field experiments were carried out in 2019 and 2020. Normal pesticide management was carried out in the field. As a result, the plant height and aboveground fresh weight and dry weight significantly increased by 23.35% at 1ng/ml ZNC, while 100ng/ml ZNC inhibited increases in aboveground fresh and dry weight compared with 0ng/ml (Table 1). This indicates that the promotion of plant growth by ZNC requires an appropriate concentration and is especially effective at low concentrations (1ng/ml) in the field.

**Table 1:** The potato growth influenced by different concentrations of ZNC through irrigation in the field.^a^

Year	Treatment (ng/ml)	Plant height (cm)	Stem diameter (cm)	On the ground	
				Fresh weight (g)	Dry weight (g)
2019	0	44.17±0.80 d	1.38±0.08 c	347.96±13.62 c	30.48±1.30 c
	1	57.53±0.55 a	1.52±0.08 a	460.97±8.26 a	38.43±0.99 a
	10	46.17±0.68 b	1.49±0.02 ab	369.35±21.33 b	33.53±0.65 b
	100	45.30±0.70 c	1.47±0.01 b	232.99±7.97 d	22.15±1.64 d
2020	0	42.70±0.10 d	1.45±0.03 a	314.62±9.73 c	29.46±1.28 c
	1	56.10±0.40 a	1.48±0.02 a	460.98±8.30 a	37.70±0.89 a
	10	45.73±0.31 b	1.53±0.02 a	369.43±21.37 b	33.46±0.89 b
	100	44.57±0.51 c	1.47±0.03 a	221.43±5.70 d	23.23±2.66 d

a Twelve plots were designed with three replicate plots per treatment. In each plot, 10 plants were randomly selected before harvest. Different letters represent significant differences compared with the values at 0ng/ml ZNC (*p*<0.05, based on one-way ANOVA analysis).

Furthermore, 1 and 10ng/ml ZNC increased potato yields by 18.83 and 7.03%, respectively, while 100ng/ml reduced the potato yield by 25.03% in 2019 (Table 2). Tuber yield per plant and single tuber weight play an important role in the components of commercial potato yield. Along with potato growth aboveground, 1ng/ml ZNC significantly increased the tuber yield per plant and the rate of large tubers with high commercial value, whereas 100ng/ml ZNC reduced these parameters by 25.03 and 32.76% in 2019, respectively, compared with 0ng/ml (Table 2). Interestingly, 10ng/ml ZNC significantly improved the rate of medium tubers, thereby increasing the rate of commercially valuable potato tubers. The same results were obtained in the next year (Table 2). These results suggested that ZNC can effectively increase marketable yields at 1 and 10ng/ml.

**Table 2:** The potato yield and its components influenced by ZNC through irrigation in the field.^a^

Year	Treatments (ng/ml)	Yield (kg/hm^2^)	Increased Production rate (%)	Number of tubers per plant	Tuber weight per plant (g)	Rate of small tubers (%)	Rate of medium tubers (%)	Rate of Large tubers (%)	Commercialization rate (%)
2019	0	44,137.00±398.24 c		4.83±0.21 a	774.33±19.18 c	11.08±1.70 b	74.39±4.86 a	14.53±4.23 bc	88.91±1.70 a
	1	52,447.60±1,990.23 a	18.83	4.73±0.12 a	920.13±34.92 a	9.14±0.96 b	63.37±2.90 c	27.49±2.16 a	90.86±0.96 a
	10	47,237.80±1,919.54 b	7.03	4.80±0.10 a	828.74±33.67 b	7.64±1.22 b	76.40±4.23 a	15.96±3.07 b	92.36±1.22 a
	100	33,090.40±390.99 d	−25.03	4.50±0.17 a	580.50±6.86 d	20.69±4.71 a	69.53±5.79 b	9.77±5.63 c	79.31±4.71 b
2020	0	43,135.70±854.47 c		4.43±0.06 b	756.77±14.99 c	10.54±1.43 b	66.92±3.42 a	22.54±3.79 b	89.46±1.43 a
	1	54,444.50±1,931.83 a	26.22	4.83±0.06 a	955.17±33.90 a	7.14±1.11 b	58.62±3.87 ab	34.24±2.83 a	92.86±1.11 a
	10	49,096.00±2,623.20 b	13.82	4.23±0.12 b	861.33±46.03 b	10.50±3.20 b	55.75±2.42 b	33.75±4.70 a	89.50±3.20 a
	100	30,831.30±1,592.30 d	−28.52	4.40±0.17 b	540.90±57.91 d	21.89±5.60 a	66.80±4.63 a	10.59±3.59 c	77.39±6.11 b

a The statistical analysis is the same as Table 1. Different letters represent significant differences compared with 0 ng/ml.

Compared to the 0ng/ml group, all ZNC treatments greatly enhanced tuber quality in 2019 and 2020. ZNC (1ng/ml) obviously improved potato quality by more than 29% in several ways, such as increasing the vitamin C, soluble protein, starch, reducing sugar, soluble sugar and dry tuber weight, and the highest increase was 125% in soluble sugar (Table 3). Therefore, ZNC improves potato quality in the field at an appropriate concentration, such as 1 or 10ng/ml.

**Table 3:** Potato quality was influenced by ZNC through irrigation in the field.^a^

Year	Treatments (ng/ml)	Vitamin C (mg/100g)	Soluble protein (mg/g)	Starch (%)	Reducing sugar (%)	Soluble sugar (%)
2019	0	20.79±0.68 c	0.17±0.01 b	12.42±0.24 c	0.26±0.01 d	3.56±0.33 d
	1	27.76±0.16 a	0.25±0.05 a	16.08±0.23 b	0.74±0.07 b	8.01±0.80 a
	10	24.55±1.04 b	0.27±0.04 a	16.41±0.18 b	0.91±0.03 a	6.28±0.04 b
	100	25.22±1.38 b	0.18±0.01 b	18.61±0.51 a	0.36±0.01 c	5.13±0.16 c
2020	0	20.00±1.56 c	0.15±0.01 c	12.98±0.84 b	0.25±0.03 d	3.00±0.13 d
	1	28.72±1.07 a	0.25±0.01 a	17.55±1.10 a	0.65±0.05 b	6.47±0.10 a
	10	25.76±1.03 b	0.26±0.01 a	17.55±0.55 a	0.83±0.09 a	5.27±0.21 b
	100	25.24±0.90 b	0.19±0.02 b	18.28±1.14 a	0.36±0.03 c	4.87±0.10 c

a In each replicate, 10 potatoes were randomly selected before harvest and used for potato quality determination. Each treatment has three replicates. Data are shown as the mean (3)±SD. Different letters represent significant differences compared with the values at 0ng/ml (*p*<0.05 based on one-way ANOVA analysis).

### Spray Application of ZNC Reduced the Occurrence of Potato Late Blight and Greatly Increased Potato Yield

**Figure 3:**
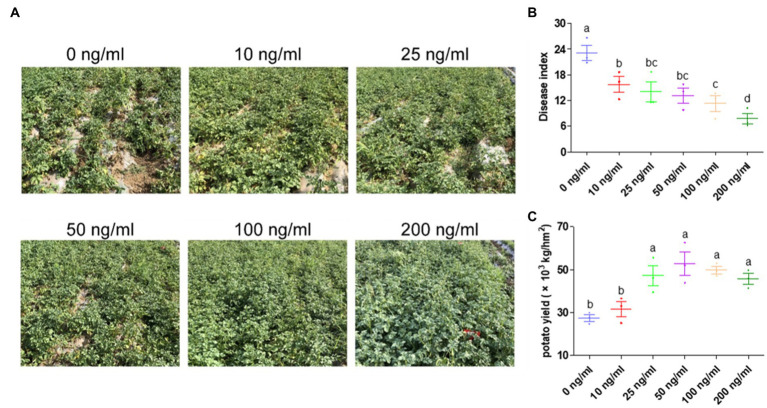
Spray application of ZNC significantly reduced the occurrence of potato late blight and greatly increased potato yield. **(A)** The performance of potato plants treated with ZNC was compared with that of the control. **(B)** Potato late blight disease index in potato plants treated with different levels of ZNC. **(C)** Field potato yield treated with different levels of ZNC. Each treatment consisted of three 36m^2^ replicate plots (6m×6m) in a randomized complete block design and 216 potato plants. Data are shown as the mean (3)±SD. Different letters represent significant differences compared with the values at 0ng/ml (*p*<0.05 based on one-way ANOVA analysis).

Both indoor and field experiments with irrigation application of ZNC showed positive effects on disease resistance and potato growth. Thus, we speculated that spray application of ZNC may contribute to the above dual functions. To verify this hypothesis, field trials utilizing spray application of ZNC were carried out in a field where late blight of potatoes often occurs. No chemical pesticides were used in the field. The performance of potato plants treated by ZNC was better than the untreated control in a ZNC dosage dependence (Figure 3A). The highest dose of ZNC (200ng/ml) showed that the control efficacy against potato late blight is 66.49% (Figure 3B). The disease severity in the 200ng/ml ZNC group was significantly lower than that in the 10–100ng/ml groups. Moreover, the yield of potatoes was significantly increased by the 25–200ng/ml ZNC treatments, among which the 50ng/ml ZNC treatment had the most obvious effect, and the yield increased by 92.19% compared with control group which led to severe production because of disease (Figure 3B).

## Discussion

### ZNC Derived From Endophytic Has Biocontrol Properties and Is Expected to Become a Key Green Agricultural Product to Enhance the Value of Agricultural Products

In the contemporary era, global demand for food continues to increase rapidly. Plant diseases, one of biotic stresses, cause about 20–40% crop yield loss every year (Ranf et al., 2011; Cabral-Pinto et al., 2019; Vega et al., 2019). The present biotic stress or plant disease control mostly depends on toxic pesticides which may pollute the environment and cause excessive residues of agricultural product (Khan et al., 2021). When plants are subjected to biotic stress, anti-disease substance accumulation occurs, and phytohormone-mediated signal pathway for disease resistance is triggered (Li et al., 2016b; Xia et al., 2019; Meng et al., 2020; Bordoloi et al., 2021). Many plant growth-promoting substances provide an alternative means to improve the utilization of agro-chemicals and enhance plant yield and quality (Asai and Shirasu, 2015; Singh et al., 2021). Plant immune inducers from microbes, like ZNC, are a useful solution for improving crop quality and yield, and an alternative for the eco-friendly control of biotic stress to meet the needs of sustainable agricultural development.

Zhinengcong is a crude ethanol extract of the endophyte *P. variotii* that can promote plant growth and enhance plant resistance to bacterial and viral pathogens indoors (Lu et al., 2019; Peng et al., 2020). ZNC also promoted potato immunity, yield, and quality, especially verified in the field (Figures 1–3 and Tables 1–3). ZNC’s increase in potato yield is closely related to auxin, which plays an important role in the regulation of plant growth and development (Hagen and Guilfoyle, 2002; Teale et al., 2006; Chandler, 2016; Li et al., 2016a).

At present, a major problem in agricultural production is the application of large quantities of fertilizer, which causes various environmental hazards (Shibata et al., 2015). Therefore, a high yield of crops can only be achieved by using nitrogen efficiently (Hirel et al., 2007). The application of ZNC with controlled-release urea has significantly increased the soil nitrogen supply in the critical period of wheat growth and development (Qin et al., 2018). A recent study demonstrates DULL NITROGEN RESPONSE1 (*DNR1*) regulates nitrogen metabolism by mediating auxin homeostasis, and OsARFs promotes nitrogen absorption rate by activating the expression of NO^3−^ metabolic related genes (Zhang et al., 2021). Combined with our research, ZNC promoted the absorption of nitrogen in potatoes, suggesting that ZNC also improves fertilizer utilization efficiency by increasing the absorption of nitrogen in plants (Figure 2F), which is beneficial for decreasing fertilizer use and reducing costs in agricultural production.

### ZNC Application Improves Disease Resistance Against Potato Late Blight Probably by Activating the PTI Pathway

The uncontrolled and indiscriminate use of chemical pesticides has resulted in pathogen drug resistance and environmental pollution with the development of modern agriculture (Lata et al, 2021). New biobased crop protection strategies are conceived due to the booming demand for environmentally satisfactory replacements for traditional pesticides (Burketová et al., 2015). Utilizing induced resistance to boost natural plant immunity is one such strategy. Plant immune inducers can alter the levels of various transcripts, proteins, and metabolites in the first stage of priming and activate plant immunity against pathogens (Alexandersson et al., 2016). In addition to plant growth promotion, ZNC could also enhance plant resistance to potato late blight indoors and in the field (Figure 1I) and by enhancing gene expression in the PTI pathway displayed by RNA-seq data (Figures 2A,B). Since the ROS burst is important for PTI (Medzhitov, 2007), 1 and 10ng/ml ZNC enhanced plant disease resistance by stimulating the PTI pathway. The extract ZNC could be perceived by plant pattern recognition receptors (PRRs) to switch on the plant defense responses, like the MAPK signaling pathway (Jeworutzki et al., 2010). The MAPK signaling pathway represents a hinge convergent module that regulates several immune responses among intracellular immune pathways (Jeworutzki et al., 2010). Some components of ZNC could induce the expression of 19 probable LRR-RLK FLS2s (Figure 2). Pathogenesis-related (PR) genes are normally regulated during the defense response. PR1 is a PR protein with anti-fungal and anti-oomycete functions that accounts for approximately 2% of all leaf proteins (Ebrahim et al., 2011). The upregulation of PR1 is used as a defense marker for salicylic acid-mediated disease resistance (Breen et al., 2017). The mechanism of disease resistance triggered by ZNC may be through the PTI pathway and a disease-resistant protein.

### ZNC Has the Advantages of Being Effective at an Extremely Low Dosage and Low Cost

In recent years, many activators have been found to induce plant defense responses or promote plant growth, and their different concentrations for application have been reported (Denoux et al., 2008). The effective concentration of ZNC for irrigation application to potato is within the range of 22.5–225mg/ha, which is significantly lower than that of other plant inducers, such as 24-epibrassinolide (750mg/ha; Ali et al., 2008), oligosaccharins (more 750mg/ha; Faoro et al., 2008), sodium nitrophenolate (more than 120g/ha; Anastassiadou et al., 2020), diethyl aminoethyl hexanoate (60g/ha; Wen et al., 2019), polyglutamic acid (more than 450g/ha; Sun et al., 2014),and aminobutyric acid (more than 200g/ha; Cohen, 2002). In addition, 2mM (approximately 4,464ng/ml) rutin resulted in good resistance to *Ralstonia solanacearum* (Yang et al., 2016). The cost per 667m^2^ of ZNC application *via* irrigation is as low as 0.6 yuan, and the cost of treating the same area with ZNC spray application is as low as 0.2 yuan, making ZNC a very cost-effective green agricultural product. ZNC has the advantages of being effective at a low dosage and low cost, and is expected to become a key green agricultural product to enhance the value of agricultural products.

## Conclusion

In our current study, the results demonstrated that ZNC had ultrahigh activity in promoting plant growth and yield and inducing disease resistance to potato late blight. ZNC promoted potato growth by inducing the expression of genes associated with auxin biosynthesis and regulating the absorption of nitrogen. ZNC promoted the transcription of PTI pathway genes to encourage potato plants to resist late blight, and these effects were verified indoors and in the field through irrigation and spray experiments. Our study will help to optimize the application of ZNC in potato production and contributes a cost-effective and environmentally friendly alternative to synthetic agro-chemicals with multipurpose use in disease management and plant productivity.

## Data Availability Statement

The datasets presented in this study can be found in online repositories. The names of the repository/repositories and accession number(s) can be found in the article/Supplementary Material.

## Author Contributions

XD conceived the idea and supervised the study. JC and BL performed the experiments and most of the analysis. XZ carried out some field experiments. XX and CZ contributed to qRT-PCR. XD, JC, BL, and YL contributed to writing of the manuscript. All authors contributed to the article and approved the submitted version.

## Funding

The authors would like to thank the Key Technology Research and Development Program of Shandong (2019JZZY020608, 2019GNC106152, and 2020CXGC010803), National Natural Science Foundation (32072500 and 31872925), Natural Science Outstanding Youth Fund of Shandong Province (JQ201807), Science and Technology Support Plan for Youth Innovation of Colleges, and Universities of Shandong Province (2019KJF023).

## Conflict of Interest

JC and XZ were employed by Shandong Pengbo Biotechnology Co., Ltd.

The remaining authors declare that the research was conducted in the absence of any commercial or financial relationships that could be construed as a potential conflict of interest.

## Publisher’s Note

All claims expressed in this article are solely those of the authors and do not necessarily represent those of their affiliated organizations, or those of the publisher, the editors and the reviewers. Any product that may be evaluated in this article, or claim that may be made by its manufacturer, is not guaranteed or endorsed by the publisher.

## Footnotes

^1^ http://www.cgmcc.net/index.html

^2^ http://www.geneontology.org

^3^ https://metacpan.org/pod/GO::TermFinder
