# Supplementary material for: Plant Endophytic Fungus Extract ZNC Improved Potato Immunity, Yield, and Quality
Source: Front Plant Sci. 2021 Sep 16;12:707256. doi: 10.3389/fpls.2021.707256 (PMC8491004; doi:10.3389/fpls.2021.707256)
Supplement: Supplementary file 1 [file Data_Sheet_1.docx]

(b)

(a)

**也应该加上差异性分析**


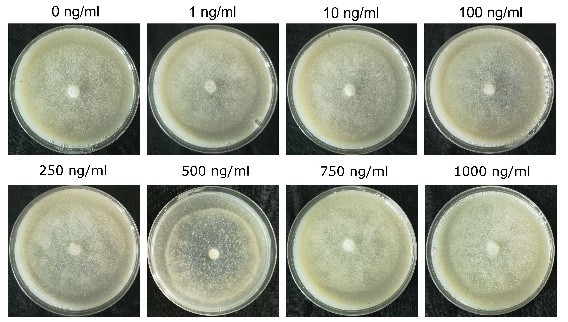


(d)

(c)

Supplementary Figure 1 | Inhibitory effect of resistance inducer ZNC on *Phytophthora infestans*. （a, b） colony sizes treated by ZNC at the following concentrations: 1, 10, 100, 250, 500, 750 and 1000 ng/ml. (c) Number of sporangia after treatment by different concentrations of ZNC. (d) Germination rate of sporangia influenced by ZNC. Data are shown as the mean (3) ± SD. Different letters represent significant differences compared with the values at 0 ng/ml ZNC (*P* < 0.05, based on Student’s *t* test)

**
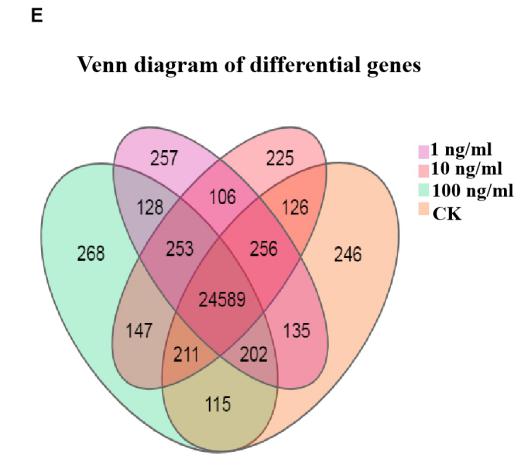

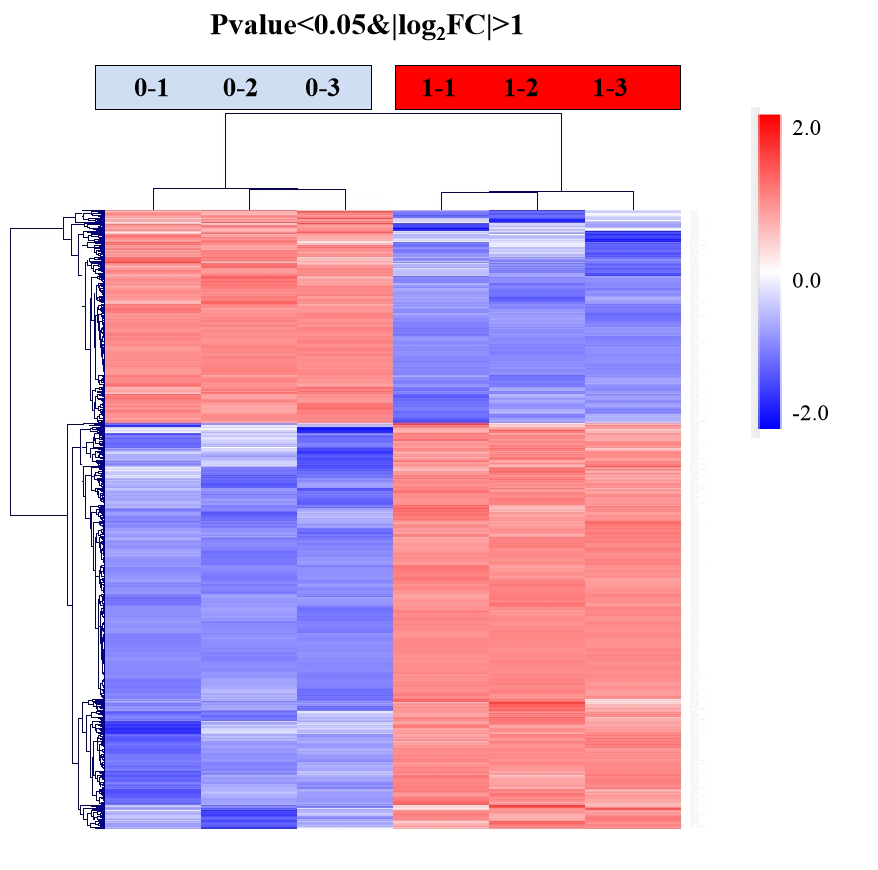

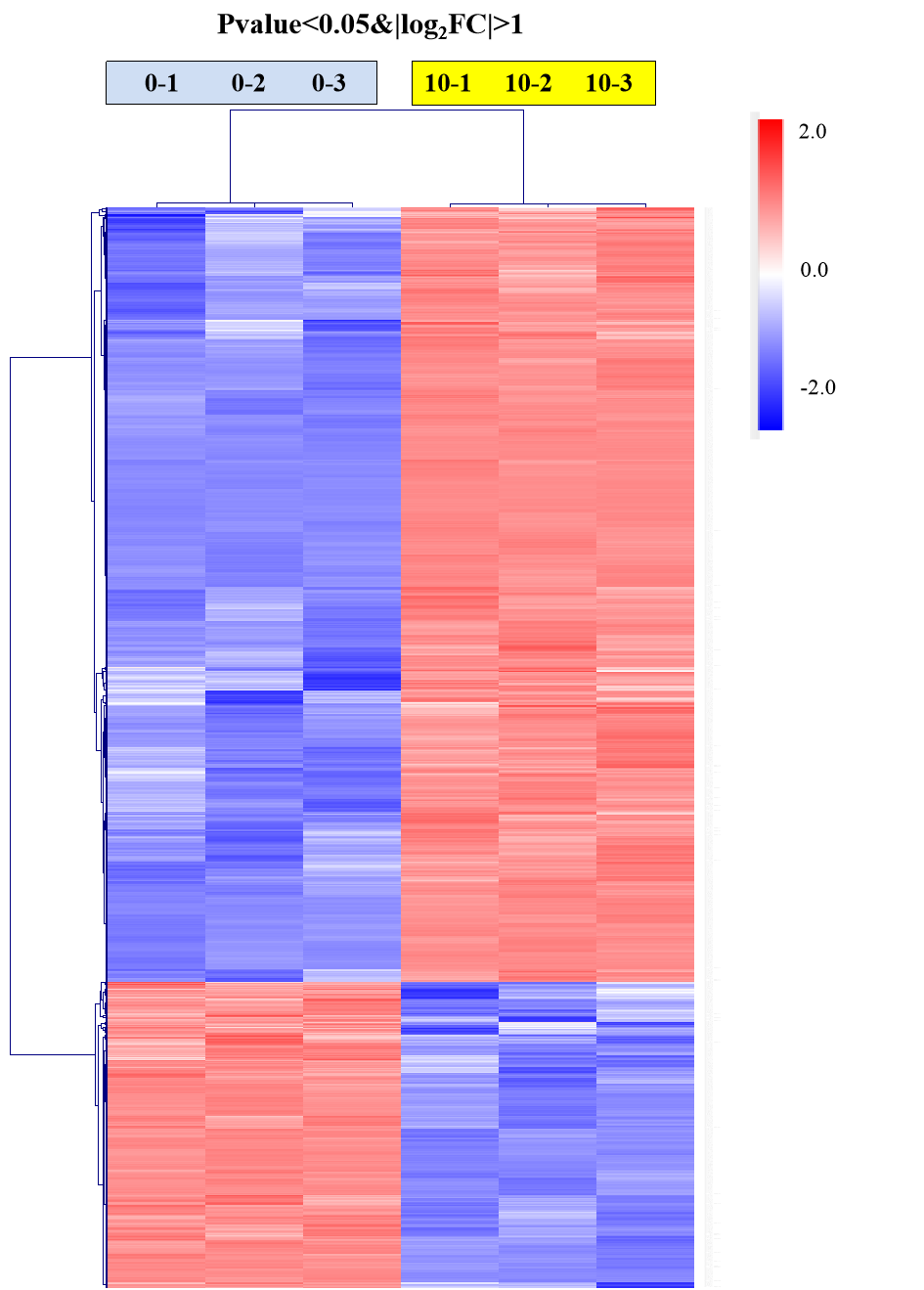

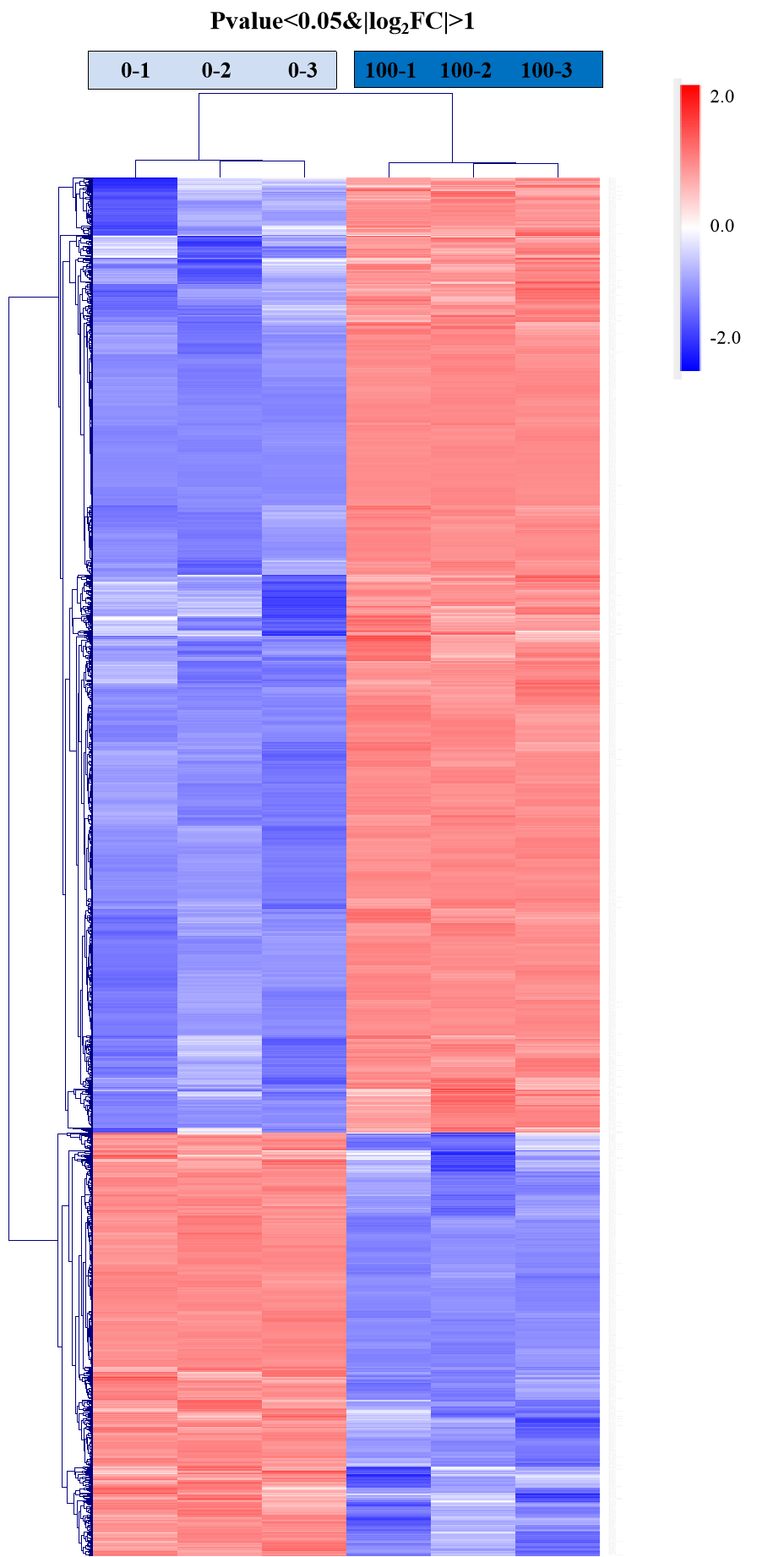
**

**(a)**

**(c)**

**(e)**

**(b)**

**(d)**

Supplementary Figure 2 | Differential gene expression profiles of ZNC-treated potatoes by RNA-seq. (a, b, c) Heatmap of differential gene expression in potato leaves treated with 1, 10 and 100 ng/ml ZNC compared with those treated at 0 ng/ml at day 40. (d) Numbers of upregulated and downregulated genes across different comparison groups. (e) Venn diagram of differentially expressed genes across different comparison groups.

**
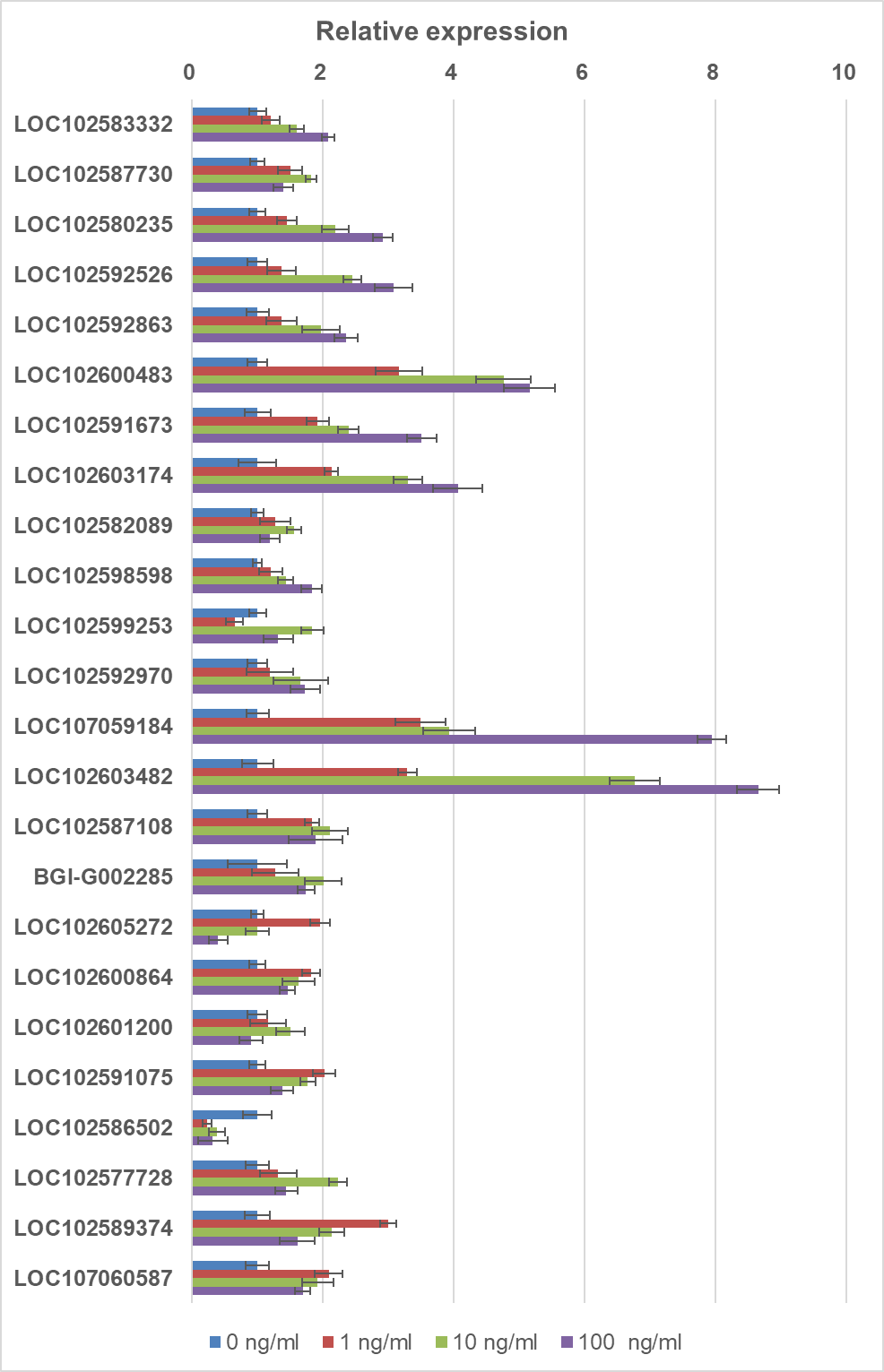
**

Supplementary Figure 3 | qRT-PCR analysis of genes in the signaling pathways subjected to ZNC treatment. Data are shown as the mean (3) ± SD. The relative expression values of mRNA were normalized to the housekeeping gene *RPN7*.

Supplementary Table 1 | List of oligonucleotide primers used in this study.

| Primers | Sequence shown in 5’→3’ orientation | Description | Amplification efficacy (%) |
| --- | --- | --- | --- |
| StEF1a-F | ATTGGAAACGGATATGCTCCA | qRT-PCR primers for AB031263 | 102.9 |
| StEF1a-R | TCCTTACCTGAACGCCTGTCA |  |  |
| StRPN7-F | TTGGGGTGTCTGAGGATTTC | qRT-PCR primers for TC203637 | 103.8 |
| StRPN7-R | CATTCTTTGCATCAGGACGA |  |  |
| LOC102583332-F | CTAATCGAGGAGTGGTACCGA | qRT-PCR primers for LOC102583332 | 90.2 |
| LOC102583332-R | GTTACTGAGGAGGGGATCTATTGA |  |  |
| LOC102587730-F | TTAGTCATAACCATCTTTGTG | qRT-PCR primers for LOC102587730 | 103.0 |
| LOC102587730-R | AGAGGCTTCCCACATAGACAA |  |  |
| LOC102580235-F | GCTGTCGTGTGGGACCTGATA | qRT-PCR primers for LOC102580235 | 104.3 |
| LOC102580235-R | ATTAGAATGAACCTCGGGGGA |  |  |
| LOC102592526-F | GCAGGACGAGGACCTAGAGAGA | qRT-PCR primers for LOC102592526 | 102.8 |
| LOC102592526-R | TCATTGAAAAAGTCTCCCATT |  |  |
| LOC102592863-F | GACCTAAAGAGACGCTACCCT | qRT-PCR primers for LOC102592863 | 100.8 |
| LOC102592863-R | TTATCACCGAGATGTTTAGTC |  |  |
| LOC102600483-F | GAACTGGAAAGAAGTGTAACC | qRT-PCR primers for LOC102600483 | 91.6 |
| LOC102600483-R | AGAAGCAGCCTTTGATGGTCC |  |  |
| LOC102591673-F | GAGAAATAGAGAGGTTGAGGG | qRT-PCR primers for LOC102591673 | 98.2 |
| LOC102591673-R | CATCTTGTTGTGTCTTTATTG |  |  |
| LOC102603174-F | TGGAAATAGAGAGGTTGAGGC | qRT-PCR primers for LOC102603174 | 97.7 |
| LOC102603174-R | TTGAATCAGCAAGTGGTGTAG |  |  |
| LOC102582089-F | GGAACAACCTTTTTGCTGGTC | qRT-PCR primers for LOC102582089 | 98.2 |
| LOC102582089-R | ATGAGCCAACATAGGAGGAAT |  |  |
| LOC102598598-F | TGGCAAATATGGTGGCAATCA | qRT-PCR primers for LOC102598598 | 99.5 |
| LOC102598598-R | CTCCACACTTATCATCCCCTG |  |  |
| LOC102599253-F | TTTGCTGAAAGAGCCAACACC | qRT-PCR primers for LOC102599253 | 103.0 |
| LOC102599253-R | GAAACCATCCTGCCTCCGAAC |  |  |
| LOC102592970-F | TCTGGTTCTGGATGACCCCTC | qRT-PCR primers for LOC102592970 | 94.7 |
| LOC102592970-R | GAGGCGATTGGCTGCTCTGTC |  |  |
| LOC107059184-F | AACAAAATGAAAGAGACGGCA | qRT-PCR primers for LOC107059184 | 92.6 |
| LOC107059184-R | TTGGGAGATGAAAGGGAAGAG |  |  |
| LOC102603482-F | ATCTCTTCCCTTTCGTCTCCC | qRT-PCR primers for LOC102603482 | 107.2 |
| LOC102603482-R | TACATTTCCCACCACCCTTTT |  |  |
| LOC102587108-F | CCCTTTGTGATCTTGGACCGC | qRT-PCR primers for LOC102587108 | 91.1 |
| LOC102587108-R | TTCGCAGAATTGTCGTGGGAG |  |  |
| BGI-G002285-F | CGCAAGTTGGAGATTAGAGAC | qRT-PCR primers for BGI-G002285 | 108.2 |
| BGI-G002285-R | GAACAATCAGGCATCCAAAGG |  |  |
| LOC102605272-F | CAGATAAAGCCGAGCCGACTA | qRT-PCR primers for LOC102605272 | 104.8 |
| LOC102605272-R | GCTTGTATCGGAGGCAGTCAG |  |  |
| LOC102600864-F | AGTTTCAATGCCTCCTTCGTT | qRT-PCR primers for LOC102600864 | 101.9 |
| LOC102600864-R | AGGACGGAAGTGAGTGATTGG |  |  |
| LOC102601200-F | ACGAAGACCACCTTCCTGTTG | qRT-PCR primers for LOC102601200 | 104.5 |
| LOC102601200-R | CAACGAAGGAGGCATTGAAAC |  |  |
| LOC102591075-F | TTATGTTACCTAGCTCGCTAC | qRT-PCR primers for LOC102591075 | 106.2 |
| LOC102591075-R | GACTTATCGGAGATTCCATTC |  |  |
| LOC102586502-F | TCGCTTACACCCTTATTCGCA | qRT-PCR primers for LOC102586502 | 103.2 |
| LOC102586502-R | TTGTATCCAGCAACCCGAAGC |  |  |
| LOC102589374-F | CAACTTGCTGACCTATCCATT | qRT-PCR primers for LOC102589374 | 102.2 |
| LOC102589374-R | AAGAAAGAGCAAGAGGTGTCA |  |  |
| LOC102577728-F | GGGCAGAGCAAATACCAGATA | qRT-PCR primers for LOC102577728 | 107.7 |
| LOC102577728-R | AATGCCAATGTTGTATGAGAT |  |  |
| LOC107060587-F | AACCAGCAGTGACTCCTATTG | qRT-PCR primers for LOC107060587 | 99.0 |
| LOC107060587-R | TTGACCAGGCTGCCCTTTTAT |  |  |

Supplementary Table 2 | Statistics of reads by different concentrations of ZNC.

| Sample | Total Raw Reads (M) | Total Clean Reads (M) | Total Clean Bases (Gb) | Clean Reads Q20 (%) | Clean Reads Q30 (%) | Clean Reads Ratio (%) | Total Clean Reads (M) | Total Mapping (%) | Uniquely Mapping (%) |
| --- | --- | --- | --- | --- | --- | --- | --- | --- | --- |
| 0 ng/ml_1 | 77.13 | 70.74 | 10.61 | 96.4 | 87.76 | 91.72 | 70.74 | 80.34 | 47.97 |
| 0 ng/ml_2 | 76.88 | 71.18 | 10.68 | 96.34 | 87.6 | 92.59 | 71.18 | 80.41 | 48.09 |
| 0 ng/ml_3 | 77.13 | 70.79 | 10.62 | 96.45 | 87.91 | 91.78 | 70.79 | 79.92 | 48.04 |
| 1 ng/ml_1 | 75.37 | 69.84 | 10.48 | 96.45 | 87.91 | 92.67 | 68.41 | 80.74 | 50.03 |
| 1 ng/ml_2 | 77.13 | 70.69 | 10.6 | 96.38 | 87.73 | 91.65 | 68.31 | 80.49 | 50.22 |
| 1 ng/ml_3 | 75.37 | 69.35 | 10.4 | 96.33 | 87.56 | 92.01 | 71.21 | 80.57 | 49.79 |
| 10 ng/ml_1 | 75.16 | 69.31 | 10.4 | 96.33 | 87.56 | 92.21 | 69.31 | 80.34 | 47.95 |
| 10 ng/ml_2 | 77.13 | 71.35 | 10.7 | 96.44 | 87.88 | 92.51 | 71.35 | 80.72 | 48.25 |
| 10 ng/ml_3 | 73.62 | 69.96 | 10.49 | 96.84 | 88.84 | 95.03 | 69.96 | 81.43 | 49.75 |
| 100 ng/ml_1 | 71.87 | 68.41 | 10.26 | 97.03 | 89.36 | 95.19 | 69.84 | 80.47 | 48.9 |
| 100 ng/ml_2 | 71.87 | 68.31 | 10.25 | 97.08 | 89.52 | 95.06 | 70.69 | 80.19 | 48.78 |
| 100 ng/ml_3 | 75.37 | 71.21 | 10.68 | 97.03 | 89.37 | 94.47 | 69.35 | 80.26 | 48.66 |

Supplementary Table 3 | The numbers of differentially expressed genes (DEG) in potato leaves treated by ZNC within the top fifth KEGG classification.

| Pathway Name | 1 ng/ml | 10 ng/ml | 100 ng/ml |
| --- | --- | --- | --- |
| Plant hormone signal transduction | 28 | 108 | 84 |
| MAPK signaling pathways | 46 | 61 | 94 |
| Plant-pathogen interaction | 32 | 50 | 84 |
| Phenylpropanoid biosynthesis | 43 | 79 | 116 |
| Starch and sucrose metabolism | 36 | 46 | 73 |
